# Supplementary material for: Cryoprotectant enables structural control of porous scaffolds for exploration of cellular mechano-responsiveness in 3D
Source: Nat Commun. 2019 Aug 2;10:3491. doi: 10.1038/s41467-019-11397-1 (PMC6677882; doi:10.1038/s41467-019-11397-1)
Supplement: Supplementary file 1 — Supplementary information [file 41467_2019_11397_MOESM1_ESM.pdf]

Supplementary Information

Cryoprotectant enables structural control of porous scaffolds for exploration of cellular mechano-responsiveness in 3D

Shumeng Jiang, Cheng Lyu et al.

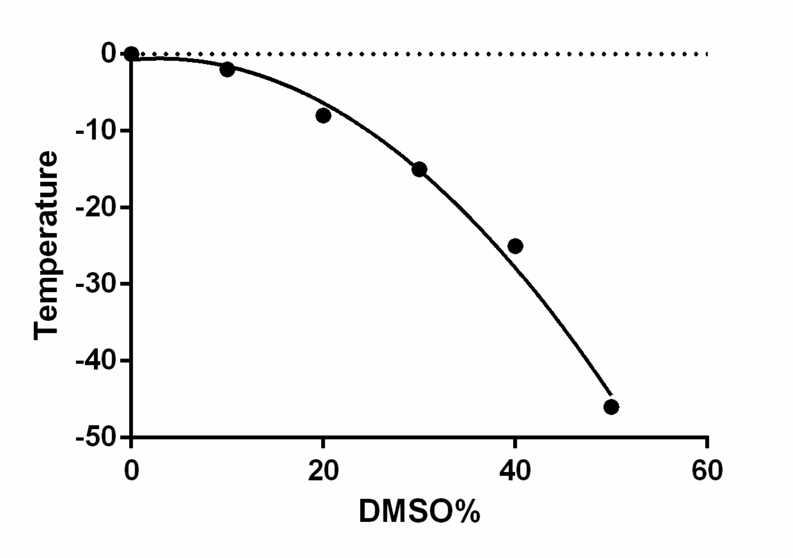

Supplementary Figure 1, Changes in the freezing point of DMSO-water mixture with different DMSO concentrations.[1]

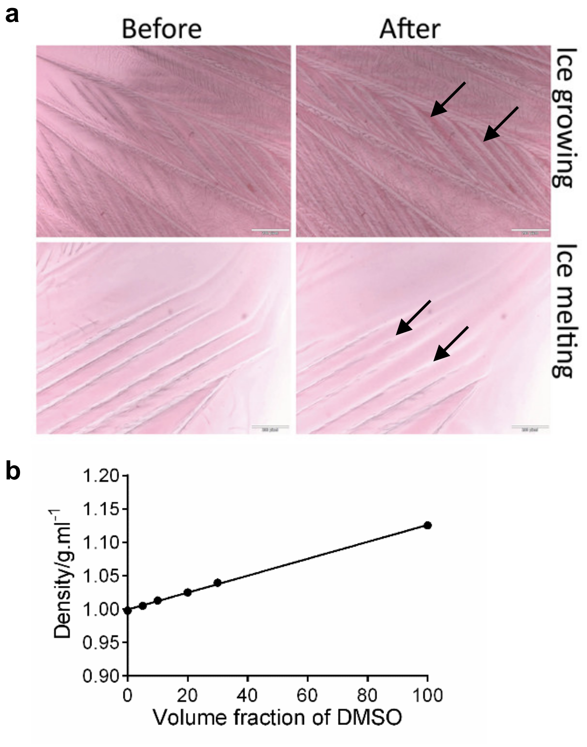

Supplementary Figure 2, DMSO accumulation during directional freezing. (a) Visualization of DMSO accumulation by oil red staining. The hydrophobic oil red dye could only dissolve in DMSO but not water thus enabling specific DMSO staining. The results showed that oil red-stained DMSO only accumulated in the non-frozen regions during ice growing, which was constituted of gaps between ice crystals, and no DMSO could be observed in the newly ice-melted regions. Scale bar: 200 μm. (b) Standard curve used for the measurement of DMSO concentration.

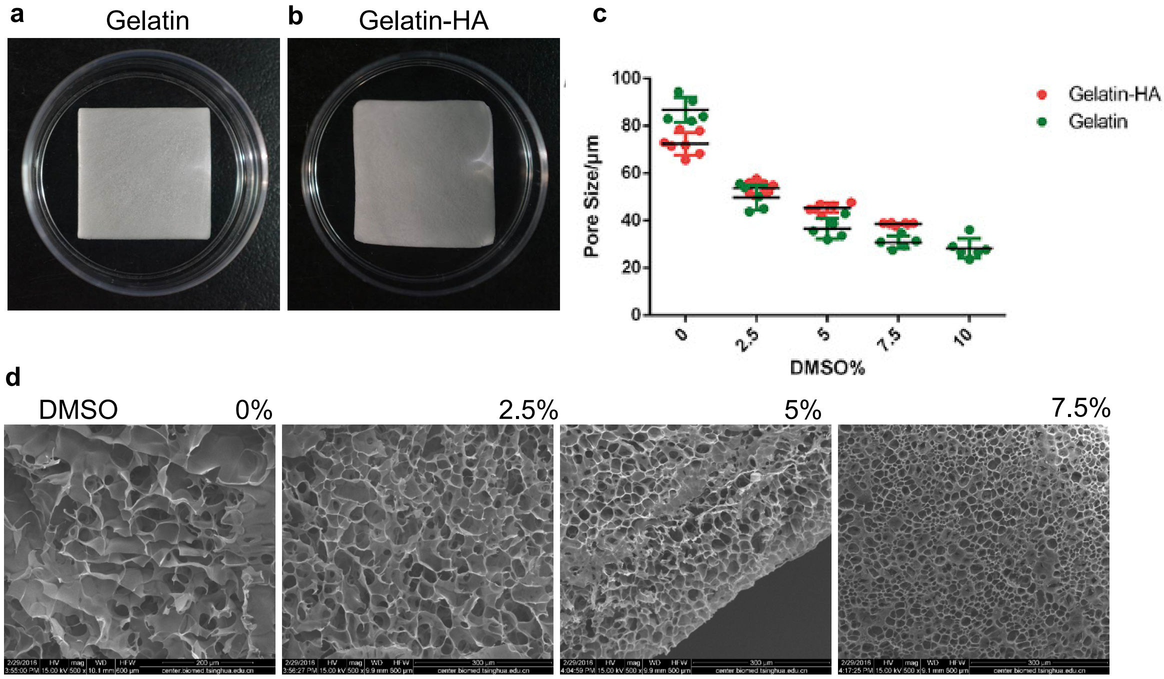

Supplementary Figure 3, Pore size controlling effects in gelatin scaffold and gelatin-HA scaffold. (a) (b) Photos of gelatin-based and gelatin-HA hybrid porous scaffolds, (c) Precise control of pore size in both gelatin-based and gelatin-HA hybrid scaffolds by different concentrations of DMSO. Data are means ± s.d. (n = 6) (d) SEM images of gelatin-HA scaffolds with well-controlled pore sizes regulated by different DMSO concentrations.

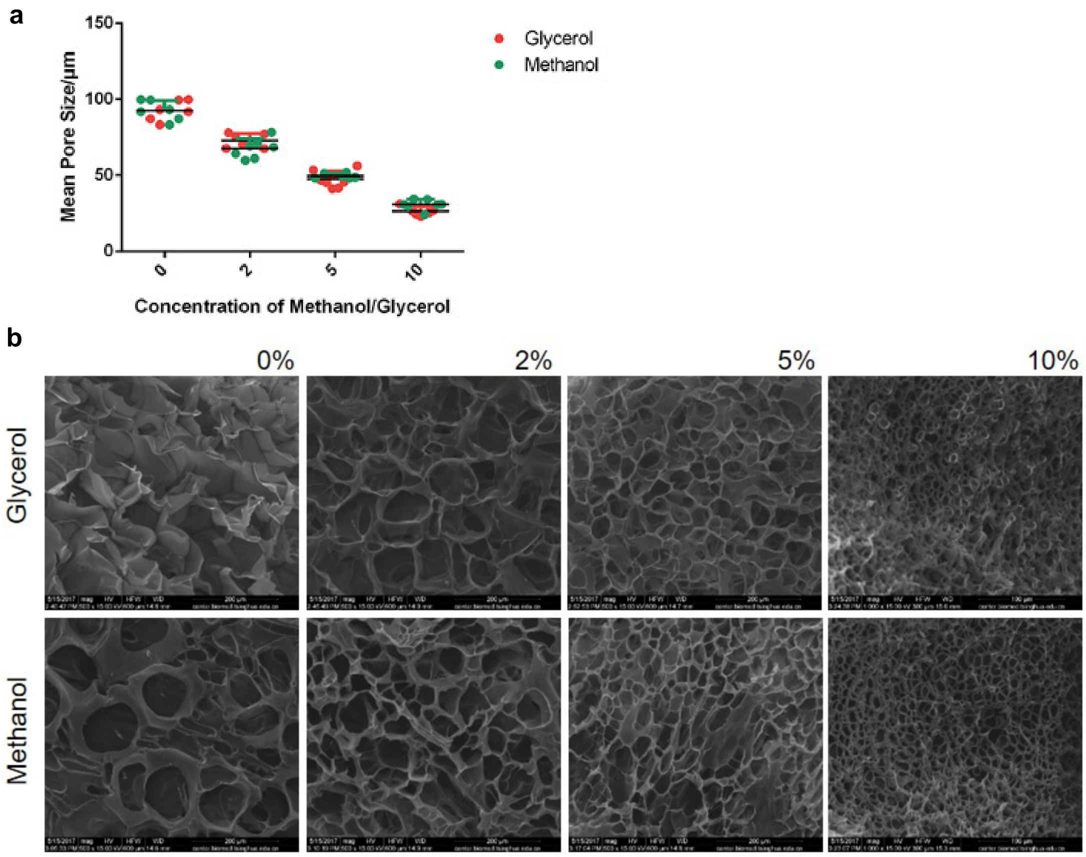

Supplementary Figure 4, Pore size regulation by other cryoprotectants. (a) Regulatory effects of other cryoprotectants such as glycerol (n = 6) and methanol (n = 7) on the pore sizes of 3D gelatin scaffolds. Data are means ± s.e.m. (b) Typical SEM images of pore structure under different glycerol and methanol concentrations showing that the pore size of gelatin scaffold could also be well regulated through controlling the concentration of these cryoprotectants.

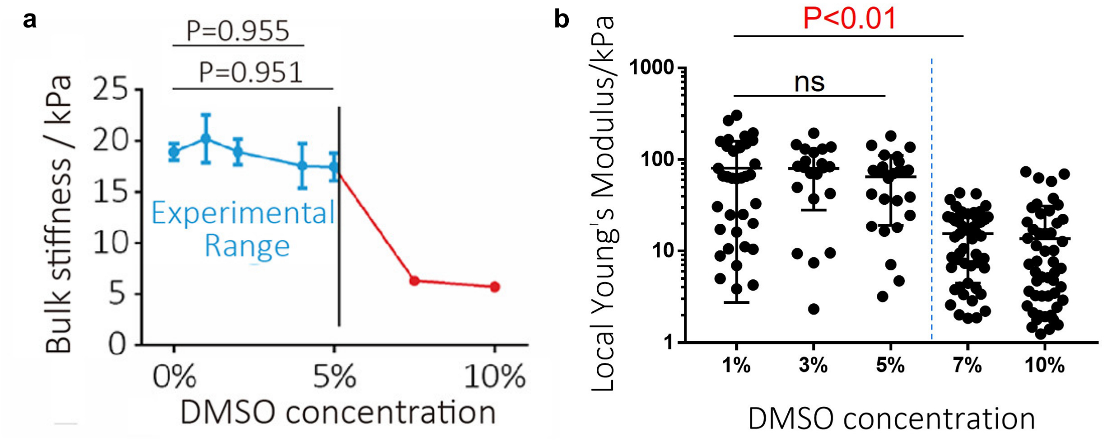

Supplementary Figure 5, DMSO concentration in the range 0%-5% did not change the stiffness of scaffolds. There was no significant change in scaffolds' Bulk stiffness (n = 3) (a) and Local stiffness (n ≥ 21) (b) with the DMSO concentration in the range of 0%-5%, which was chosen as the 'Experimental range' to eliminate the influence of DMSO on stiffness of the scaffolds. Data are means ± s.d. (ANOVA)

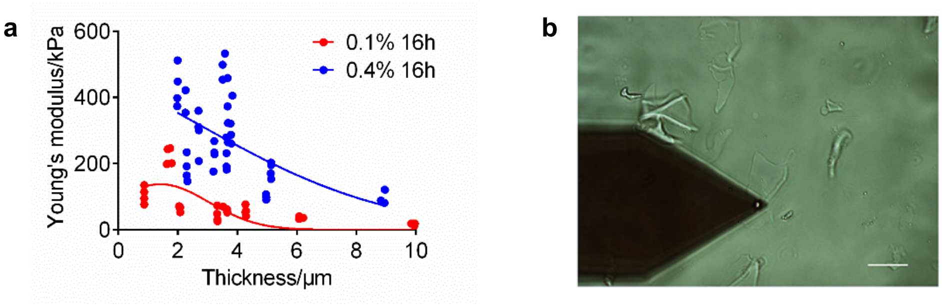

Supplementary Figure 6, Correlation between the thickness and stiffness of scaffold fragments. (a) Correlation between the thickness and stiffness of scaffold fragments. (b) Image of scaffold fragments and AFM tip under light microscope. Scale bar: 50 μm.

During AFM test, we found the difference in thickness of scaffold fragments could lead to great variance in measured value. Therefore, we examined the thickness of every fragment while we measured its stiffness using AFM. It showed that with the increase in fragment thickness, the measured stiffness was decreased. Therefore, we only included the stiffness measurement for those fragments with thickness ranging from 2 to 4 μm. When the fragment was too thin, the stiffness may rise because of the influence of the glass substrate. However, when the fragment thickness was too high, the fragments might not attach well on the glass substrates, leading to unstable measurement.

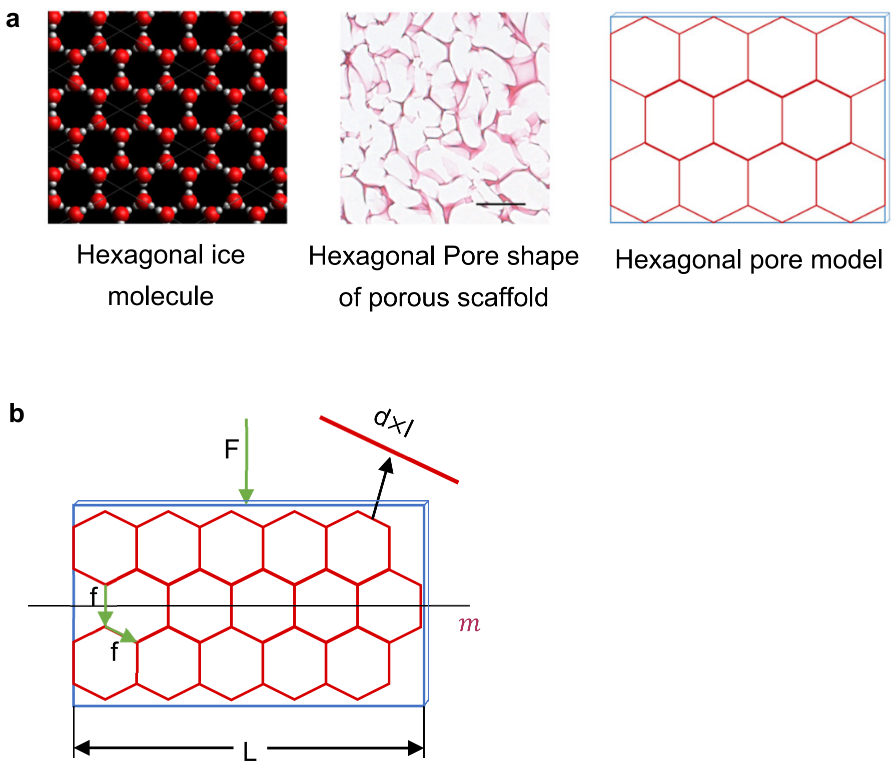

Supplementary Figure 7, A mathematical model to predict the correlation between local and bulk stiffness. (a) The hexagonal ice molecule resulted in hexagonal pore structure, which inspired us to develop a hexagonal model to calculate the correlation between bulk stiffness and local stiffness in the porous scaffolds. Scale bar: 50 μm (b) Hexagonal mathematic model to verify the experimental results measured in AFM test. We defined related parameters in the model as illustrated in the image: The F represents the bulk force. The L represents the length of the section. The f represents the force on every fiber. The m represents the number of fibers in the horizontal direction. The d and l represent the width and length of a single fiber.

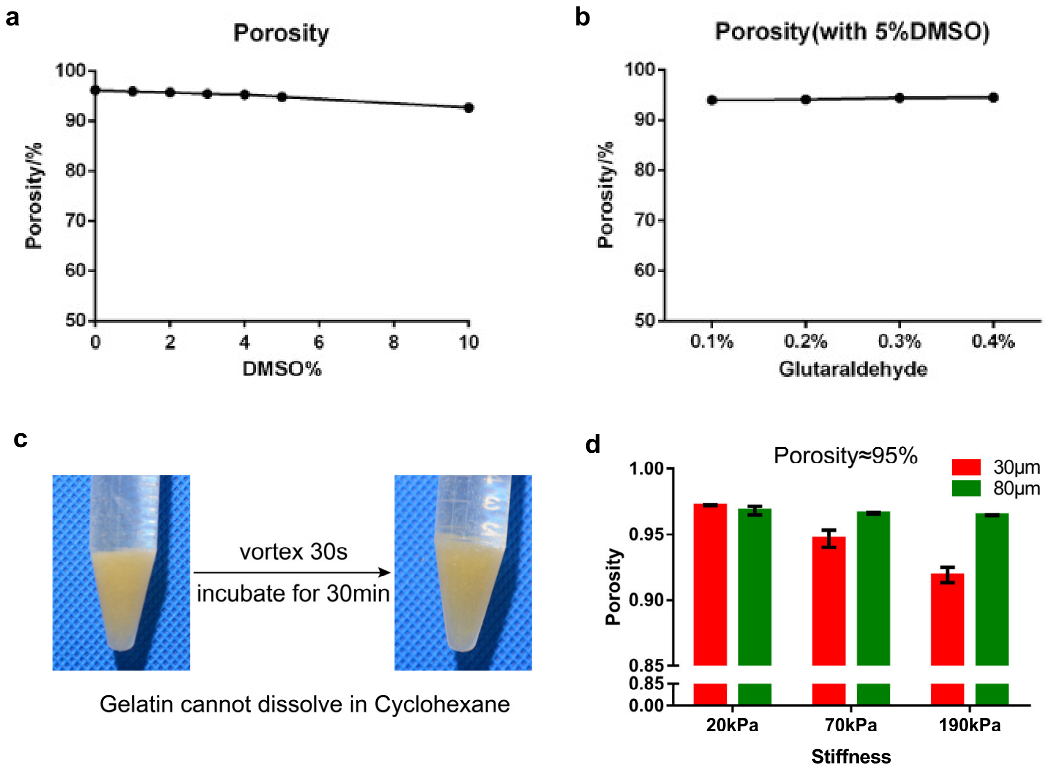

Supplementary Figure 8, Porosity of scaffolds was constant among scaffolds with different pore sizes and stiffness. Porosity of the scaffolds was not influenced by either (a) DMSO-based pore size regulation or (b) GA-based stiffness regulation (n ≥ 3). (c) (d)Porosity of the scaffolds measured by cyclohexane, as a pore filling reagent. Gelatin is almost insoluble in cyclohexane. The porosity can be calculated by the following formula:

$$Porosity=\frac{V_{pores}}{V_{dry}+V_{pores}}=\frac{(W_{absorbed}-W_{dry})/\rho_{solvent}}{W_{dry}/\rho_{gelatin}+(W_{absorbed}-W_{dry})/\rho_{solvent}}$$

The density of cyclohexane ($\rho_{solvent}$) was 0.779 g/cm^3, and the density of the dry scaffold was approximately the density of gelatin($\rho_{gelatin}$, 1.037 g/cm^3).The absorbed ($W_{absorbed}$) and dry (W_{dry}) weight of scaffolds could be measured which led to the conclusion that the porosity, ~95%, did not exhibit significant difference among all the scaffolds. The conclusion is consistent with the previous porosity estimation based on water (n ≥ 2). Data are means ± s.e.m.

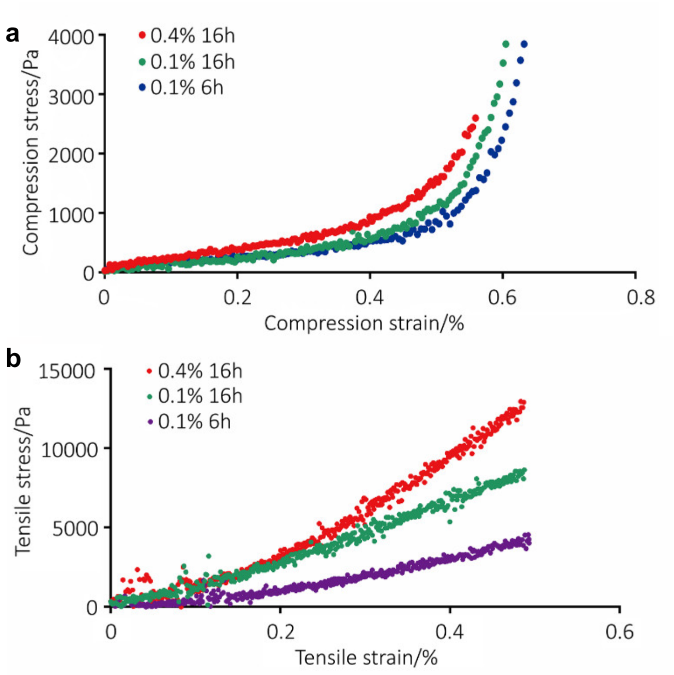

Supplementary Figure 9, Stress-strain curves of compression (a) and tensile (b) test of the porous scaffolds.

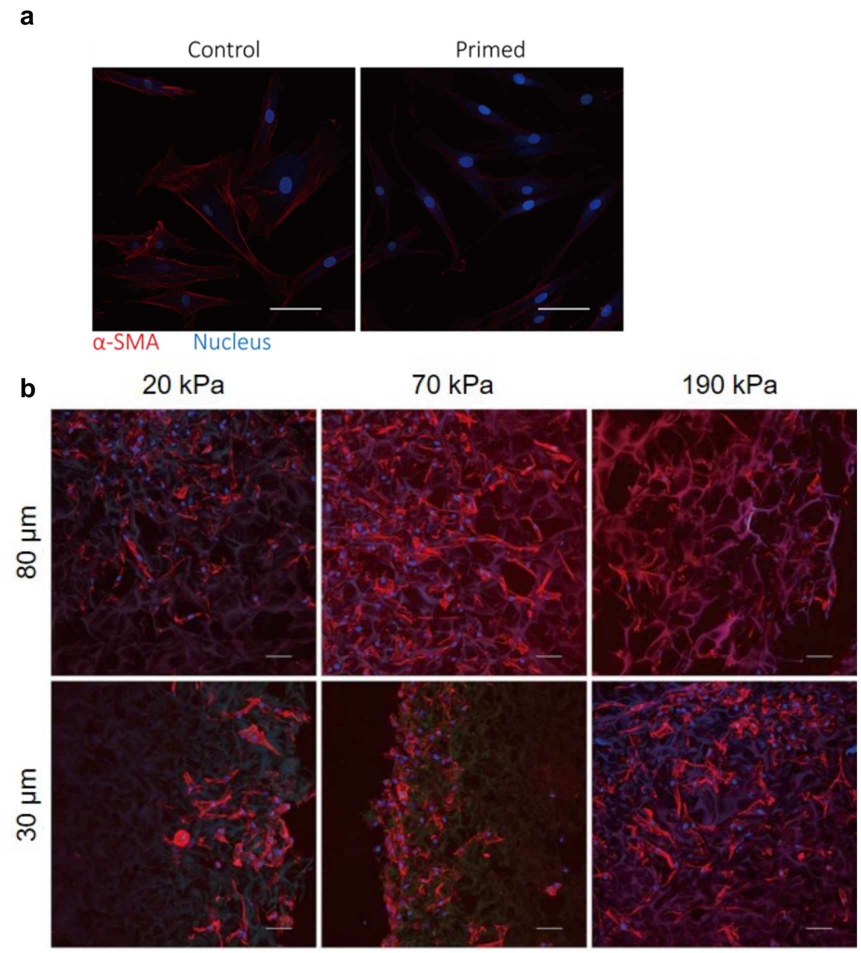

Supplementary Figure 10, HDFs pre-priming was important to maintain non-activated phenotype of HDFs. (a) HDFs expanded in normal culture dishes exhibited an activated phenotype with high αSMA expression, which could be inactivated by being primed on soft PEG-based hydrogel substrate. (b) High αSMA expression would be observed in all scaffolds if non-primed HDFs were seeded into the different 3D porous scaffolds directly, indicating no mechano-responsiveness to the scaffold properties and potential mechanical memory of HDF cultured on rigid culture-dish. Scale bar: 50 µm.

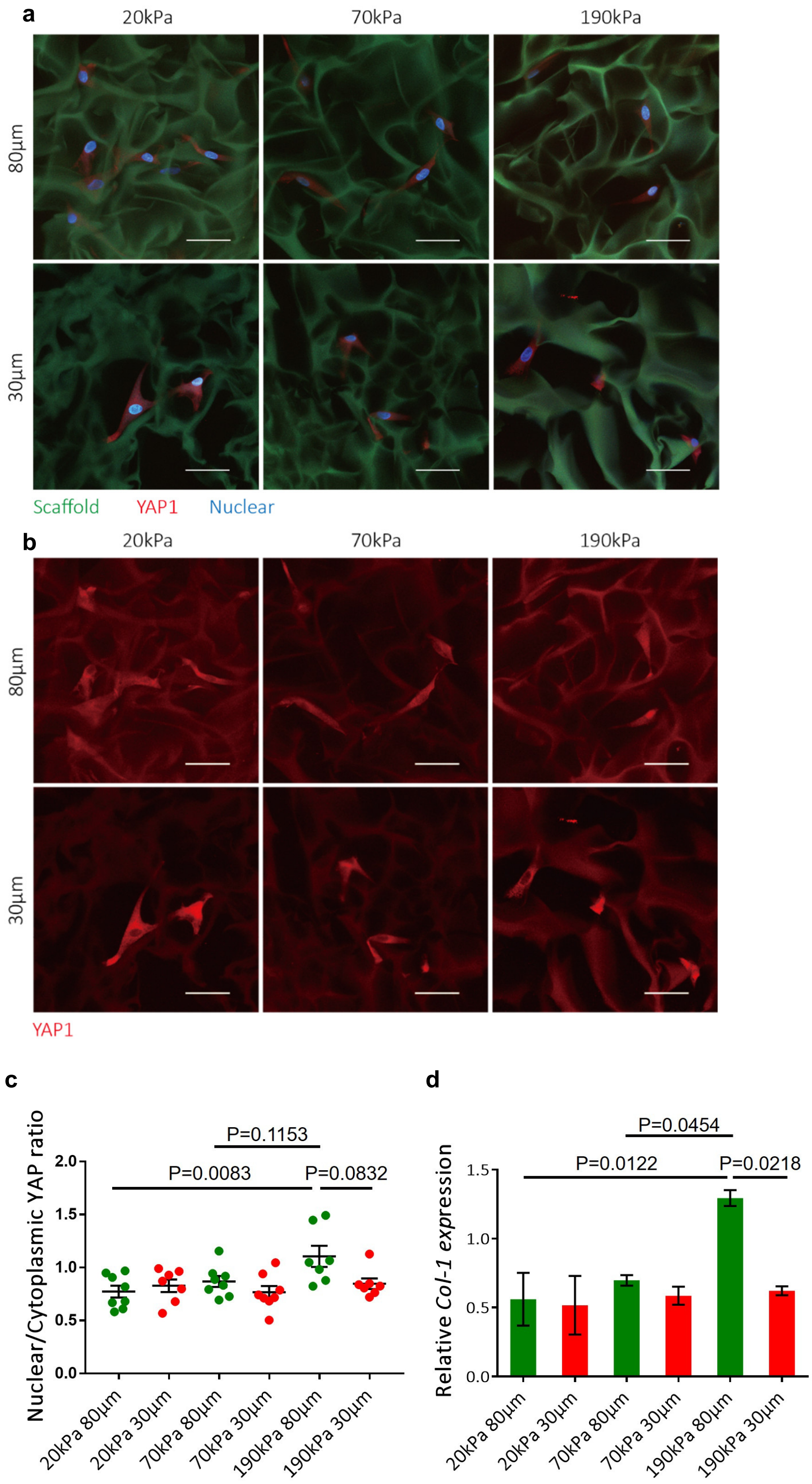

Supplementary Figure 11, Scaffolds regulated HDF phenotype through YAP localization. (a) (b) (c) images of YAP1 showed the significant cytoplasmic localization in small pore scaffold and nucleus localization in 190 kPa, 80 µm group. (n = 7) Scale bar: 50 µm. (d) qPCR characterized *Col-1* expression of HDFs. (n = 3) Data are means ± s.e.m. (ANOVA)

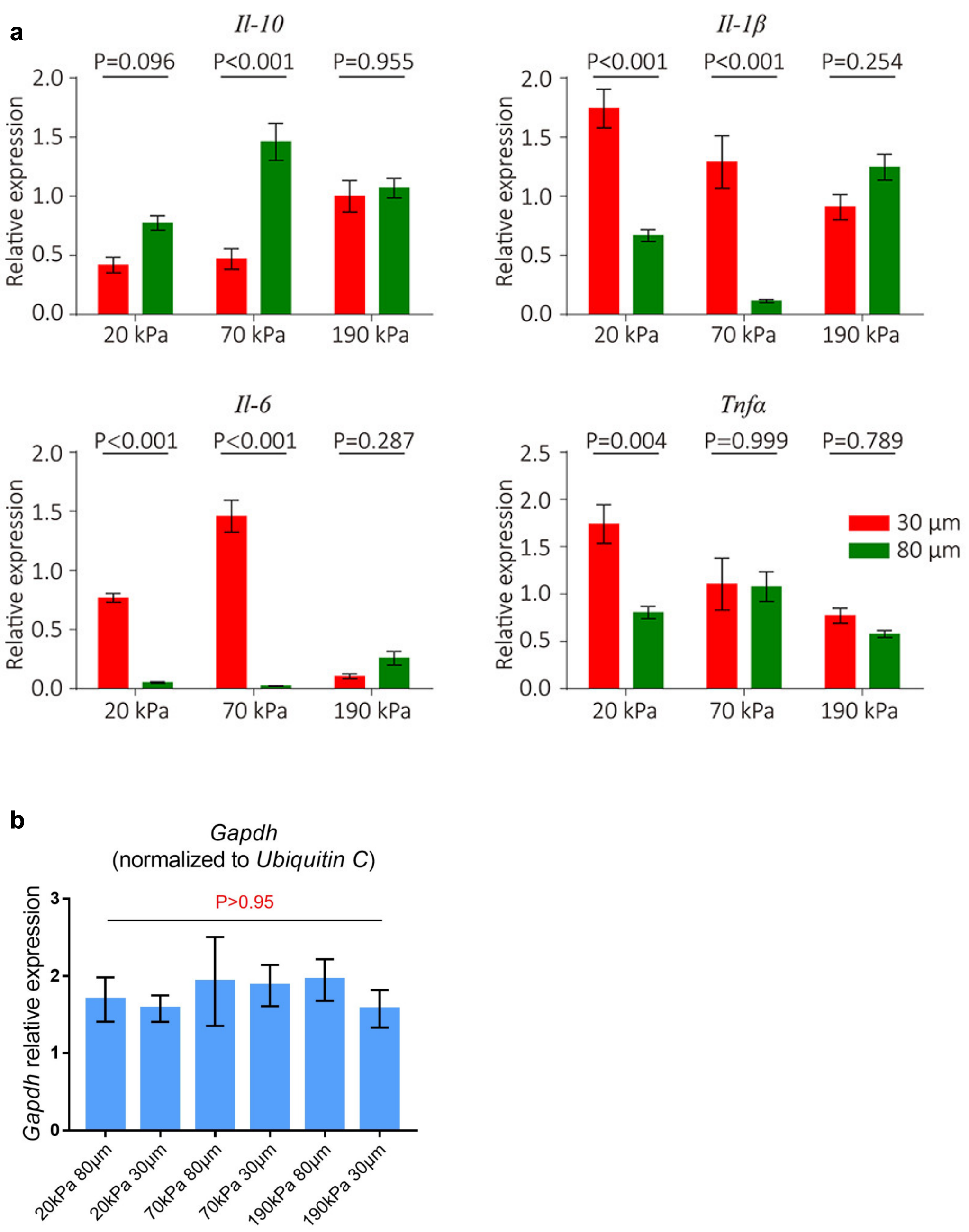

Supplementary Figure 12, M1 / M2 gene expression levels were regulated by the physical properties of the scaffolds. (a) Gene expression level of pro-inflammatory cytokines (*Il-6*, *Il-1β*, *Tnf-α*) and anti-inflammatory cytokine (*Il-10*). (Normalized to 2D-culture control). The expression of these genes was closely correlated with iNOS / Argianse-1 expression. Specifically, higher expression of *Il-6*, *Il-1β* and *Tnf-α* and lower expression of *Il-10* were only observed for BMDMs within scaffolds with soft and small pores. (n = 3) (b) *GAPDH* expression was verified to be stable for scaffolds with different pore sizes and stiffness when normalized to another housekeeping gene, *Ubiquitin c*.[2] (n = 4) Data are means ± s.e.m. (ANOVA)

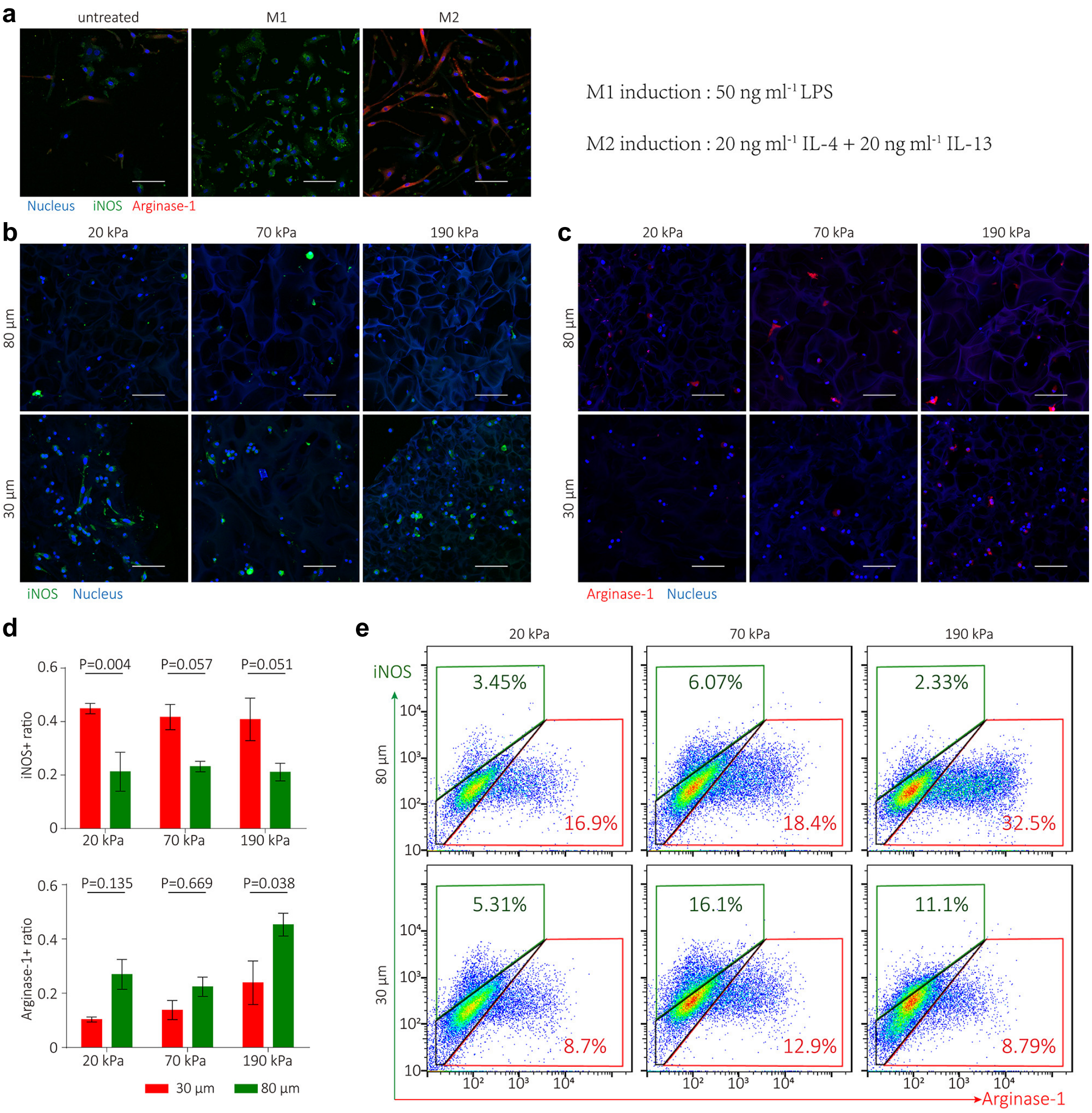

Supplementary Figure 13, Macrophages phenotype regulated by scaffolds with additional M1 inducing factors. (a) M1 / M2 induction in 2D culture system. M1 macrophages were induced by 50 ng ml^{-1} LPS for 3 days. M2 macrophages were induced by 20 ng ml^{-1} IL-4 and 20 ng ml^{-1} IL-13 for 3 days. (b-e) BMDM primed in scaffolds were induced by 50 ng ml^{-1} LPS for 3 days and were stained by (b)iNOS for M1 and (c)Argianse-1 for M2. (d) Statistical analysis of iNOS+ / Arginase-1+ cells in scaffolds. ($n \geq 3$) (e) Flow cytometry analysis of iNOS+ / Arginase-1+ cells in scaffolds. Scale bar: 50 µm. Data are means ± s.e.m. (ANOVA)

In 2D-culture group, increased iNOS (M1 marker) expression and increased Arginase 1(M2 marker) could be observed with M1 and M2 induction respectively. When BMDM were cultured in scaffolds with M1 induction, overall increase in iNOS level and decrease in Arginase-1 level were observed. More iNOS+ cells could be observed in scaffolds with small (i.e. 30 µm) pores, regardless of pore stiffness. In contrast, most Arginase-1+ cells could be observed within stiff and large pores (i.e. 190 kPa, 80 µm), whereas few Arginase-1+ cells could be observed within soft and small pores (i.e. 20 kPa, 30 µm). Consistent with the immunofluorescent staining, flow cytometry analysis showed that there were highest ratio of Arginase-1+ cells (32.5%) and lowest ratio of iNOS+ cells (2.33%) in scaffolds with stiff and large pores (190 kPa, 80 µm) and low level of Arginase-1+ cells

(8.7%) in scaffolds with soft and small pores (i.e. 20 kPa, 30 μm).

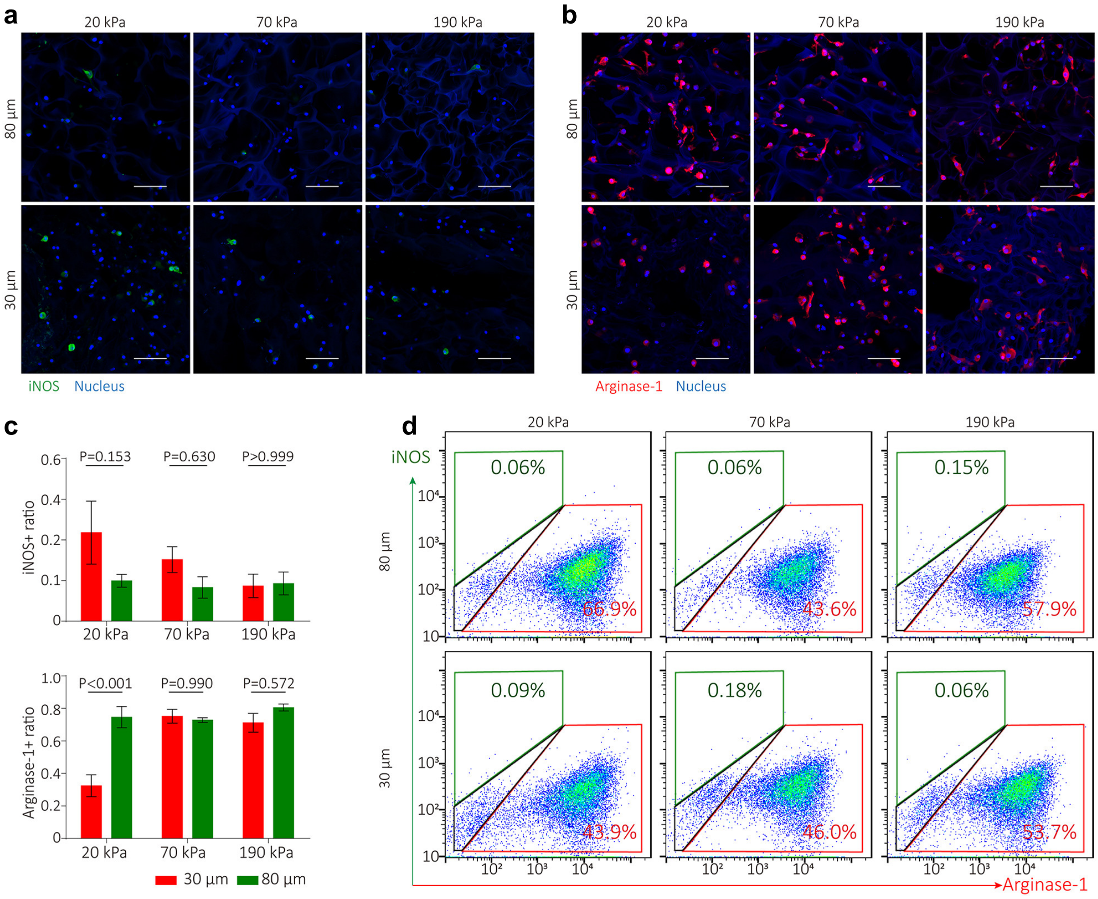

Supplementary Figure 14, Macrophages phenotype regulated by scaffolds with additional M2 inducing factors. BMDM primed on scaffolds were induced by 20 ng ml^{-1} IL-4 and 20 ng ml^{-1} IL-13 for 3 days and were stained by (a)iNOS for M1 and (b)Argianse-1 for M2. (c) Statistical analysis of iNOS+ / Arginase-1+ cells in scaffolds. (n ≥ 3) (d) Flow cytometry analysis of iNOS+ / Arginase-1+ cells in scaffolds. Scale bar: 50 μm. Data are means ± s.e.m. (ANOVA)

When BMDM were cultured in scaffolds with M2 induction, sharp decrease in iNOS (M1) level and increase in Arginase-1(M2) level were observed for all the groups. Still, cells in scaffolds with soft and small pores (i.e. 20 kPa, 30 μm) showed the highest iNOS level with ~22% iNOS+ cells, while few iNOS+ cells could be observed with stiffer and larger pores (190 kPa, 80 μm). In terms of arginase-1 expression, cells in scaffolds with soft and small pores (i.e. 20 kPa, 30 μm) exhibited the lowest arginase-1 level (~32%), while rest of the groups all exhibited high aginase-1 level (~70%). Consistent with the immuofluorescent staining, flow cytometry results showed that there were few iNOS+ cells (<0.2%) and more Arginase-1+ cells in scaffolds with larger pores (i.e. 66.9% in 20 kPa, 80 μm vs 43.9% in 20 kPa, 30 μm; 57.9% in 190 kPa, 80 μm vs 53.7% in 190 kPa, 30 μm).

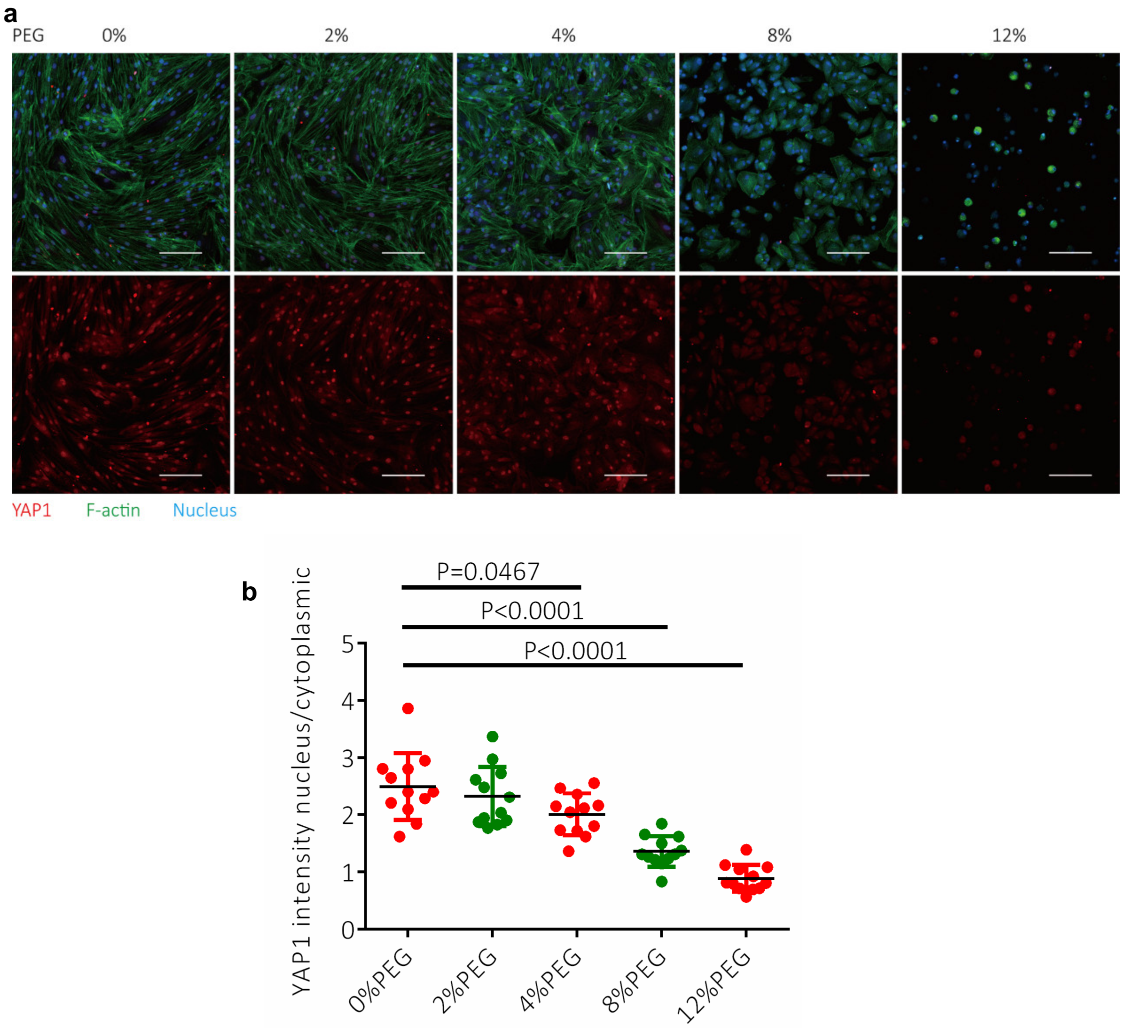

Supplementary Figure 15, High osmotic pressure caused by PEG could induce the cytoplasmic translation of YAP1 from the nucleus. (a) Confocal images of HDF stained by YAP 1(red), F-actin, (green). Scale bar: 100 µm. (b) Statistical analysis of YAP1 localization. Data are means ± s.d. (n = 12, ANOVA)

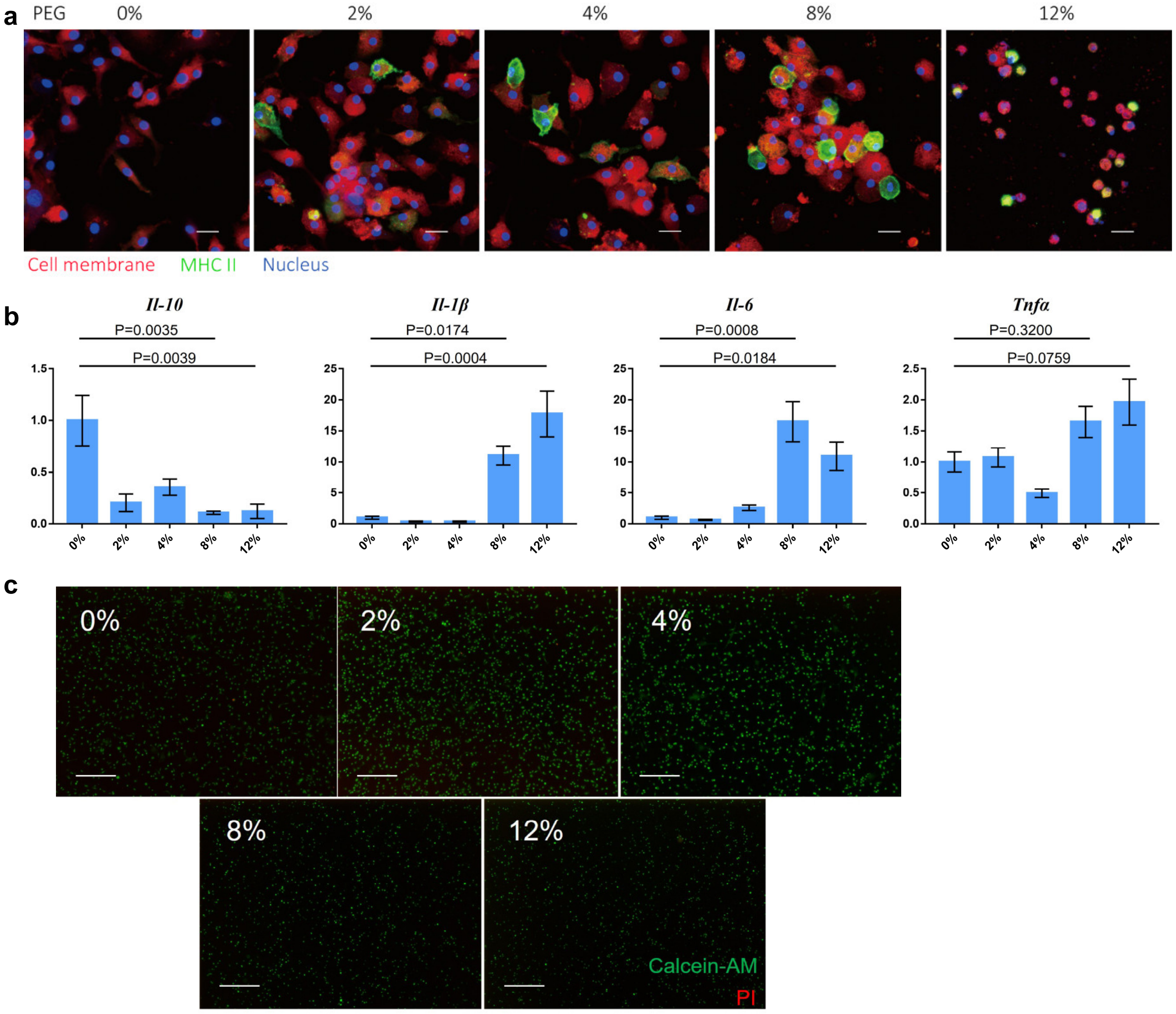

Supplementary Figure 16, High osmotic pressure caused by PEG could regulate phenotype of macrophages. (a) High resolution fluorescent images showed the gradual rounded-up of macrophages when osmotic pressure was increased, along with (b) reduced gene expression of the anti-inflammatory cytokine *Il-10* and increased gene expression of the pro-inflammatory cytokines *Il-1b*, *Il-6* and *Tnf-α*. Scale bar: 20 µm. Data are means ± s.e.m. (n = 3, ANOVA) (c) Live/dead staining showed that PEG did not exert significant cytotoxicity to macrophages. Live cells were stained by Calcein-AM(green). Dead cells were stained by PI(red). Scale bar: 200 µm

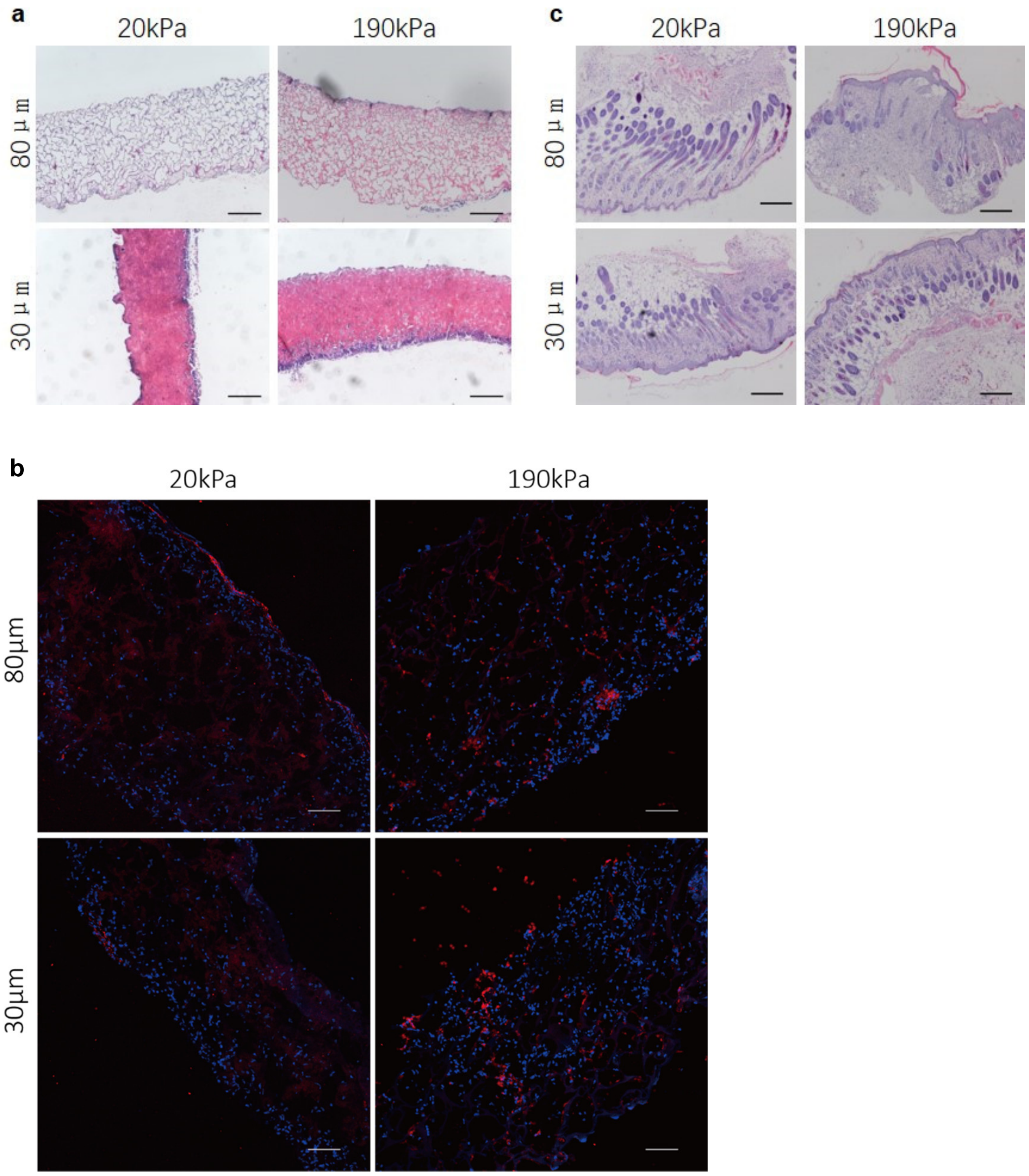

Supplementary Figure 17, Cell infiltration in implanted scaffolds in the skin wound model. (a) after 2 days' implantation, few cells were found to infiltrate into the scaffolds for all groups. Scale bar: 200 μm. (b) Staining of αSMA positive cells infiltrated into the scaffolds after 4 days of implantation. There was relatively few αSMA positive cells insides at this early stage. Scale bar: 100 μm. (c) 8 days after implantation, sufficient regeneration with skin accessories could be achieved in all groups and scaffolds may degrade or shrink to small size during wound healing. Scale bar: 200 μm

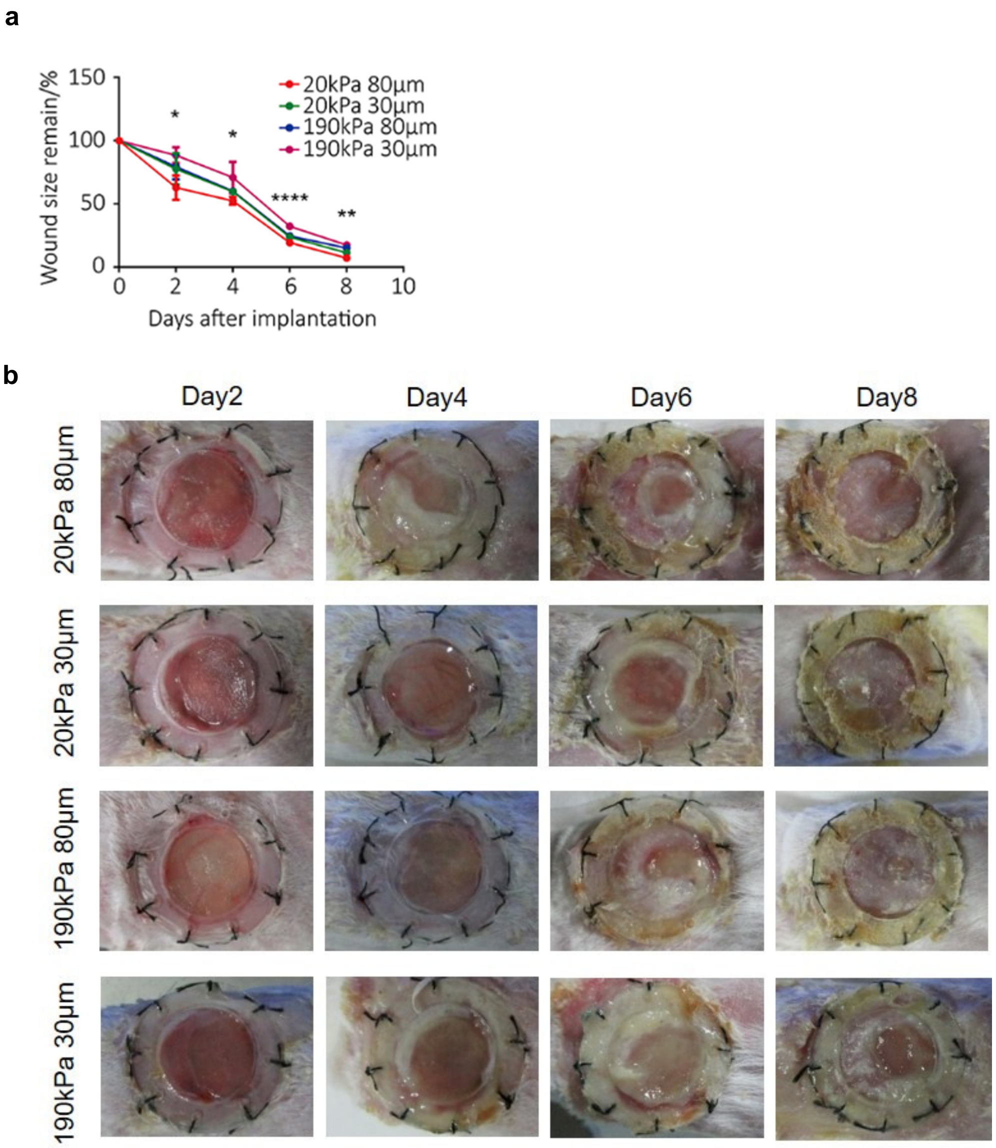

Supplementary Figure 18, Wound closure upon scaffold implantation. (a) Physical properties of the implanted scaffolds led to different wound closure rate in the mouse skin wound healing model. The statistical significance here represents the differences between the 20 kPa, 80 μm group and the 190 kPa, 30 μm group. Data are means ± s.e.m. (n = 3, ANOVA) (b) Images of typical wound at different time after being implanted of scaffolds with varied stiffness and pore size.

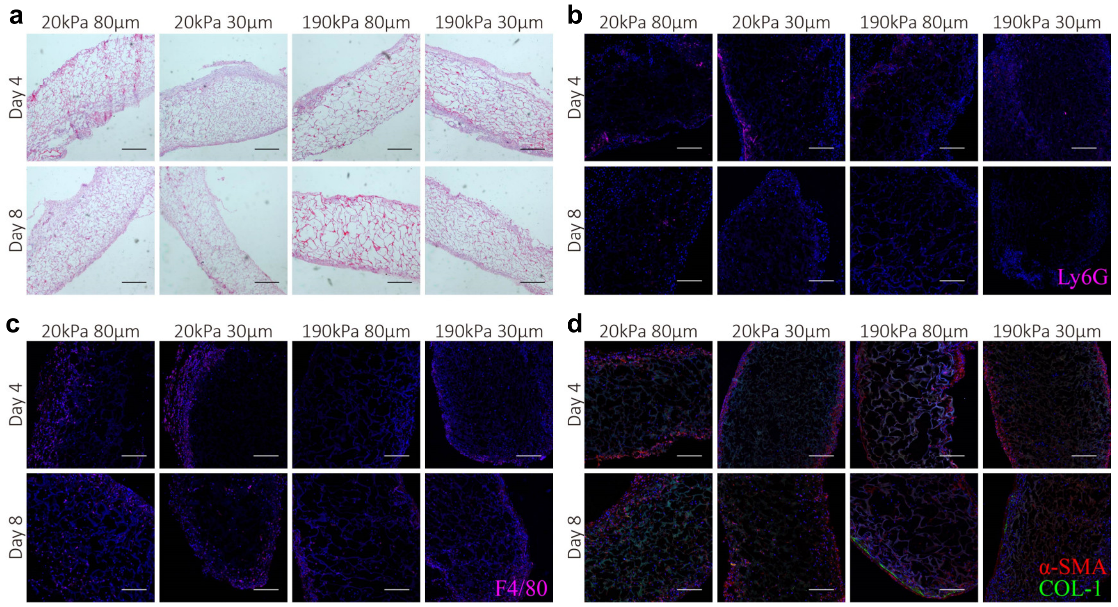

Supplementary Figure 19, Foreign body responses evaluation in a subcutaneous model. Scaffolds with different pore size and stiffness were implanted subcutaneously under back skin of 8-week-old balb/c mice and stained for (a) HE, Scale bar: 200 µm (b) neutrophils (Ly6G), (c) macrophages(F4/80) and (d) Myofibroblasts (α-SMA and COL-1). Scale bar: 100 µm.

A great number of macrophages(F4/80) and neutrophils(Ly6G) were found to infiltrate into scaffolds with small pores (i.e. 30 µm) on day 4, especially in scaffolds with small and soft pores (i.e. 20 kPa, 30 µm). While, few macrophages and neutrophils could be observed in scaffolds with stiff pores, indicating a pro-inflammatory regulation by scaffolds with soft and small pores in early stage of foreign body response. Meanwhile, infiltration of the activated fibroblasts showed no significant difference among different groups. However, collagen deposition, indicating fibrotic responses, could be observed on the edge on implanted scaffolds with stiff pores, especially in scaffolds with stiff and large pores (i.e. 190 kPa, 80 µm). These results indicated that scaffolds with small and soft pores (i.e. 20 kPa, 30 µm) could induce pro-inflammatory response shortly after implantation. In contrast, scaffolds with large and stiff pores (i.e. 190 kPa, 80 µm) would reduce inflammatory response in early stage but may induce fibrotic responses which facilitates the pro-wound healing effect.

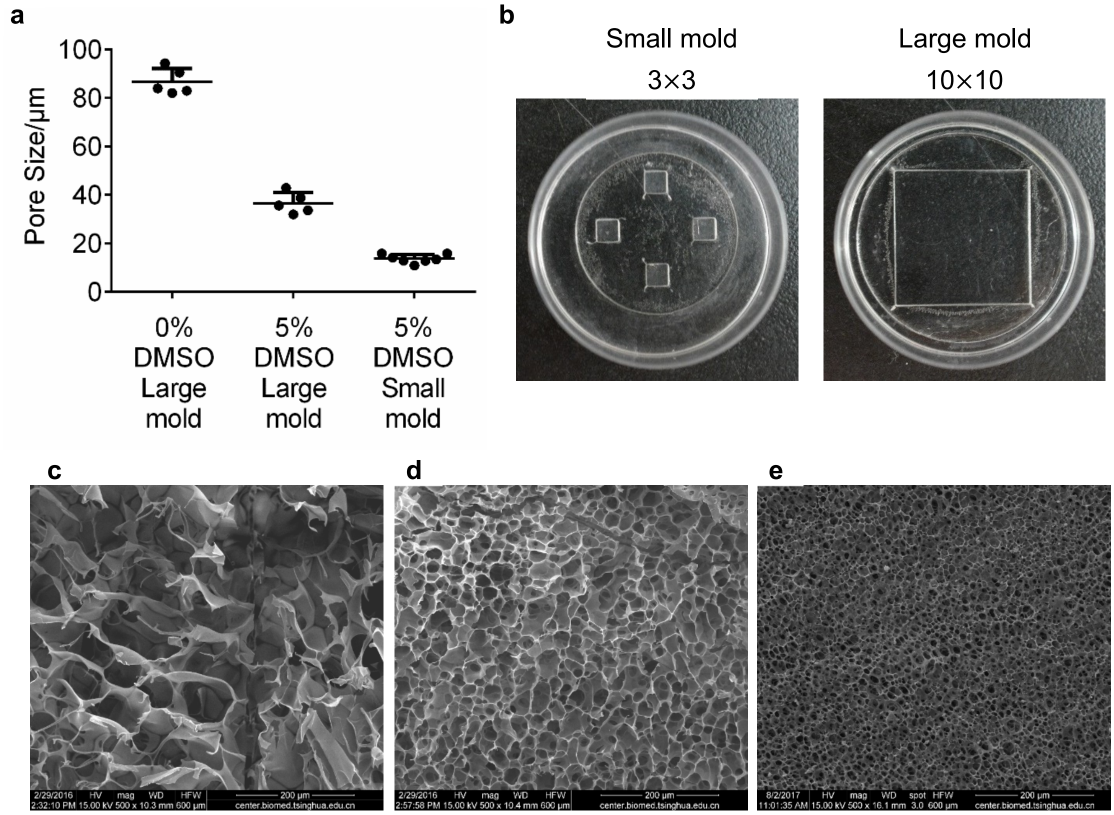

Supplementary Figure 20, The cooling rate of the cryogelation process could be regulated by altering the size of the mold. (a) pore size controlling effects of the mold size and DMSO. Data are means ± s.d. (n = 6) (b) smaller mode : 3×3 $(\mathrm{mm})^2$ large mold : 10×10 $(\mathrm{mm})^2$. (c) (d) (e) SEM images of pore structure of the scaffolds fabricated by using (c)large mold with 0% DMSO, (d) large mold with 5% DMSO, and (e) small mold with 5% DMSO.

The result showed that smaller mold may be applied to prepare scaffolds with smaller pore size due to faster cooling rate, which had been proved to be important for pore size control of cryogelated scaffold in previous studies.[3]

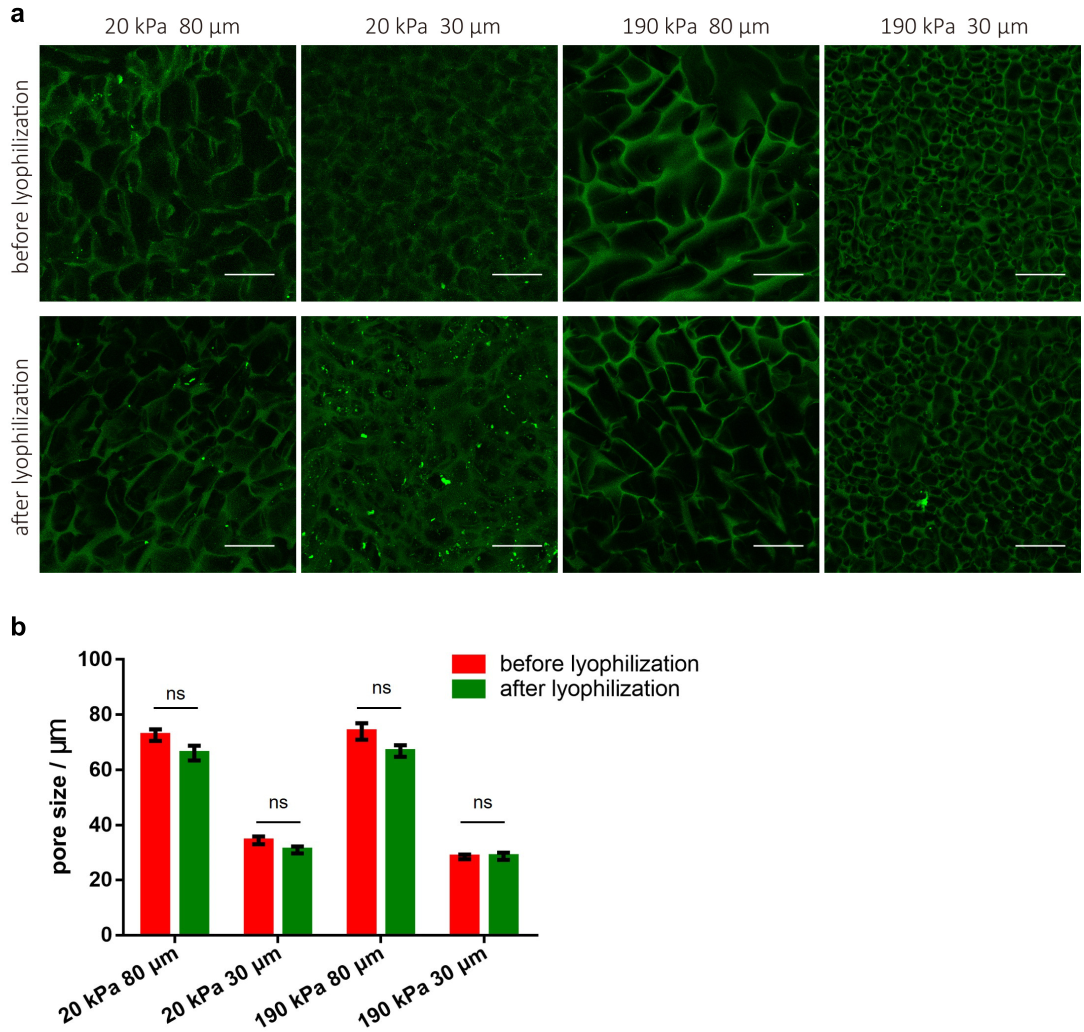

Supplementary Figure 21, Pore structure was not affected by washing, refreezing and lyophilization process. (a) confocal fluorescent images of the scaffolds before and after refreezing and lyophilization. The images were taken with 30 frames with z steps of 2 µm and made maximum projection. Scale bar: 50 µm (b)Pore sizes were analyzed by image J. Data are means ± s.e.m. ($n > 30$, $p>0.05$, two-tailed Student's t-test)

Since the pores within scaffolds have been already fully formed before refreezing, the ice crystal formation during refreezing is expected to be confined by scaffolds' porous structure, thus the refreezing and lyophilization could not alter the pore structure.

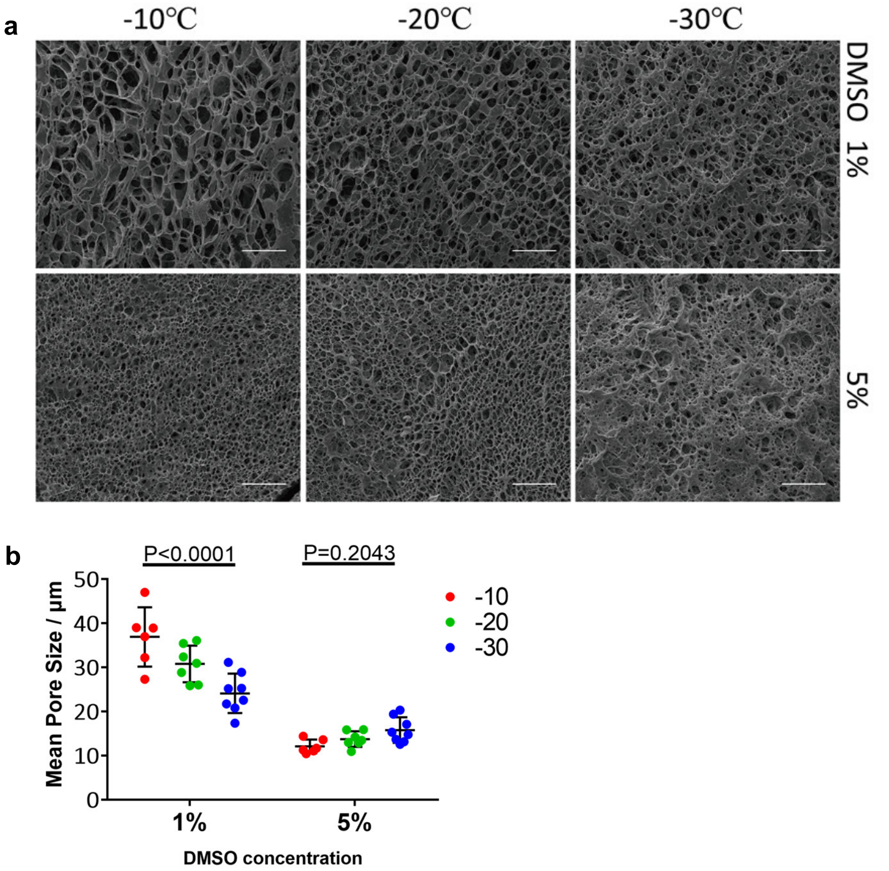

Supplementary Figure 22, DMSO regulation on pore size is affected by reaction temperature. (a) Typical SEM images of pore structure fabricated under different reaction temperatures and DMSO concentrations. (b) Pore size quantification of the SEM images. The results showed that 1) Higher concentration of DMSO led to smaller pore size; 2) Lower reaction temperature led to smaller pore sizes; and 3) The pore regulatory effect of DMSO was more prominent in higher reaction temperature. Scale bar: 50 μm. Data are means ± s.d. ($n \geqslant 6$, ANOVA)

1% DMSO exerted insignificant effects on the temperature control of pore size during cryogelaton, where lower temperature resulted in faster ice crystal formations thus smaller pore sizes. Under 5% DMSO, the enriched DMSO concentrations required to reach the freeze point were increased as the temperatures dropped (i.e. ~-10°C for 20% DMSO, ~-20°C for 35% DMSO, and ~-30°C for 45% DMSO), indicating extended duration for ice crystal formation under lower temperatures thus resulting in larger pores. This discrepancy in pore formation effects governed by both the temperature and DMSO concentration further proved our theory regarding cryoprotectant-regulated ice crystal growth.

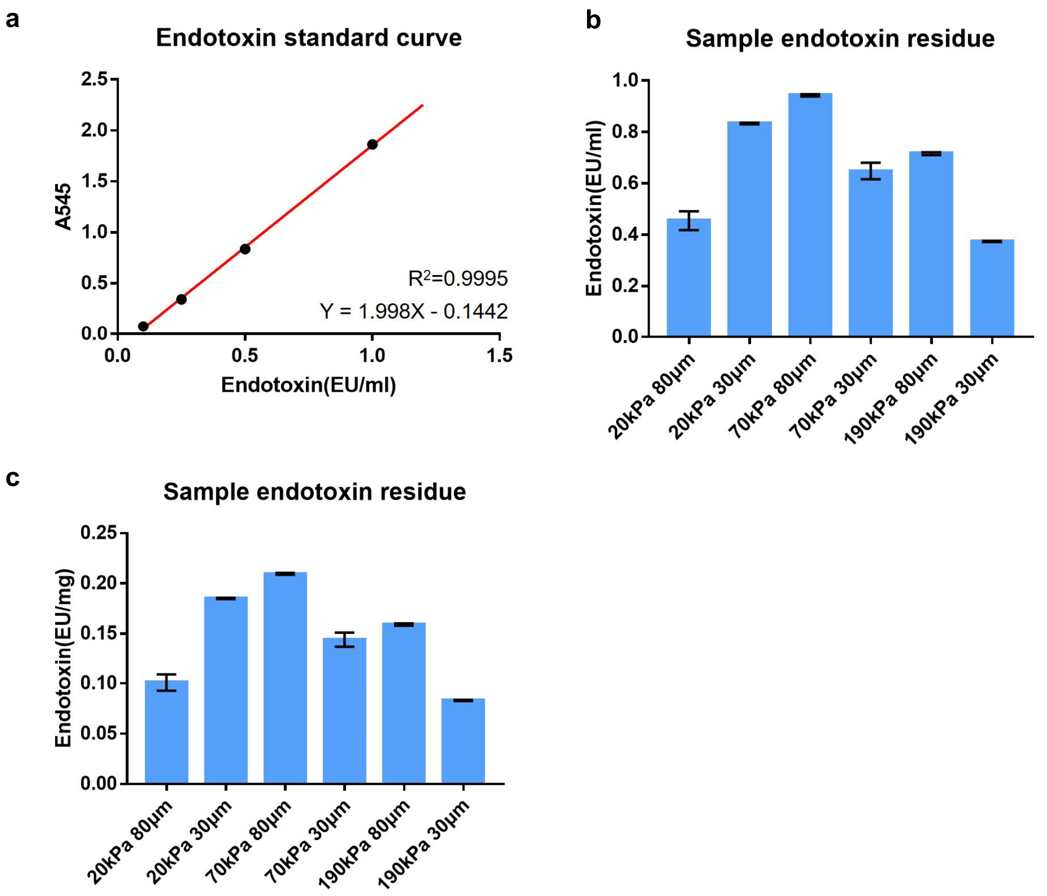

Supplementary Figure 23, Endotoxin eluates from scaffolds.

(a) Standard curve used for endotoxin detection. (b) endotoxin eluates from different scaffolds were prepared by incubation of the scaffolds in cell culture condition (37°C, 5%CO2) for 3 days and were detected by endotoxin kit (Genescript, L00350). (c) Endotoxin residue normalized to scaffolds weight. Data are means ± s.e.m. (n = 4)

Results showed that endotoxin eluates are all < 1 EU ml^{-1}, and endotoxin content of the scaffolds is below 0.2 EU mg^{-1}.

Supplementary video 1, Enrichment of DMSO during ice crystal growth indicated by hydrophobic oil red;

Supplementary video 2, Decrease in the average color intensity of the melting DMSO-water solution during ice melting.

Supplementary video 3, The regulatory effect of DMSO on ice crystals size formed in 0% DMSO-water solution;

Supplementary video 4, The regulatory effect of DMSO on ice crystals size formed in 10% DMSO-water solution;

Supplementary video 5, The regulatory effect of DMSO on ice crystals size formed in 30% DMSO-water solution;

Supplementary video 6, 3D reconstructed confocal images of HDFs in scaffolds with stiff and large pores (190 kPa, 80 μm);

Supplementary video 7, 3D reconstructed confocal images of HDFs in scaffolds with soft and large pores (20 kPa, 80 μm);

Supplementary video 8, 3D reconstructed confocal images of HDFs in scaffolds with stiff and small pores (190 kPa, 30 μm);

Supplementary video 9, 3D reconstructed confocal images of HDFs in scaffolds with soft and small pores (20 kPa, 30 μm);

References:

1. Havemeyer, R. N. Freezing point curve of dimethyl sulfoxide—water solutions. Journal of Pharmaceutical Sciences 55, 851-853 (1966).
2. Kalagara, R. et al. Identification of stable reference genes for lipopolysaccharide-stimulated macrophage gene expression studies. 1, 1-8, Biology Methods and Protocols (2016).
3. O’Brien, F. J. et al. Influence of freezing rate on pore structure in freeze-dried collagen-GAG scaffolds. Biomaterials 25, 1077-1086 (2004).
